# Investigating hope in oral health promotion for adolescents: an exploratory study based on observations at the dental clinic

**DOI:** 10.3389/froh.2024.1303933

**Published:** 2024-02-16

**Authors:** Arefe Jasbi, Kari Sand, Zoe Marshman, Marikken Høiseth

**Affiliations:** ^1^ Department of Design, Faculty of Architecture and Design, Norwegian University of Science and Technology, Trondheim, Norway; ^2^ Department of Health Research, SINTEF Digital, Trondheim, Norway; ^3^ School of Clinical Dentistry, University of Sheffield, Sheffield, United Kingdom

**Keywords:** adolescents, hope, oral health, dental health professionals, participatory research, well-being

## Abstract

**Introduction:**

Maintaining well-being is crucial, especially in challenging conditions, considering the common public health issue of dental caries. Within the context of adolescent oral health, this research explores the techniques employed by dental professionals to potentially foster hope -a positive manner that promotes well-being- in adolescents during consultations, opening a window into the realm of patient engagement and well-being.

**Materials and methods:**

Data were collected through observations conducted at public dental clinics in Norway, with the participation of three dental professionals and four adolescents between the ages of 12 and 15 years. The data were analyzed using thematic analysis. Practices were observed from dental professionals in their interactions with adolescents, which align with features of hope.

**Result:**

Three core themes were identified: (1) bonding strategies; (2) verbal and non-verbal strategies for creating positive relationships; and (3) adolescents' empowerment in dental consultations.

**Conclusion:**

Although a new concept within oral health promotion, it seems that dental professionals in this study were observed to be facilitating hope in adolescents when they were providing their dental care. Consideration should be given to the potential for future approaches to be developed for use in dental consultations to facilitate hope strategically. While these approaches are likely to contribute to improving patient-centredness, consideration is needed of challenges and barriers to their implementation.

## 1 Introduction

Dental caries among children and adolescents remains a significant public health issue (1). It can have adverse effects on a child's quality of life, academic performance, and overall cognitive and psychosocial development (2). Moreover, the risk of dental caries tends to increase as individuals reach the age of 12 (1) due to diet (3), potential declines in oral hygiene practices (4, 5), and independence in seeking or avoiding dental care (6). Despite progress being made in children's oral health, data from Norway indicates that two-thirds of 18-year-olds have experienced dental caries (7), and dental erosion affects 38% of 16-year-olds (8).

Notably, dental anxiety affects approximately 20% of the global population, particularly among children and adolescents aged 3–18 years old (9, 10), and around 13% of adolescents in Norway (11, 12). Individuals with anxiety experience negative thoughts and feelings, which affect their behaviors within the dental clinic and their ability to cope with dental procedures (10, 13). For example, adolescents report anticipating adverse outcomes (like expecting pain or clinical mistakes), reliving past traumatic dental experiences through memories or nightmares, and deliberately avoiding dental visits through tactics like deception or negotiation (10). These negative perceptions of dentistry can go beyond dental anxiety alone (14). For many people, dental visits are often equated with discomfort, pain, or even shame and guilt about the state of their oral health (15).

The phenomenon of hope has been studied extensively in social sciences and applied to health and healthcare settings (16, 17). Hope, which is a multidimensional phenomenon referring to a combination of positive expectations, goals, and thoughts about the future, has been previously investigated in relation to positive health outcomes for adolescents. For example, Berg et al., (18) conducted a study involving 48 participants to investigate the relationship between hope and adherence to pediatric asthma treatment, finding that hope significantly predicted treatment adherence. Hagen et al. (19) similarly discovered that children with higher levels of hope exhibited fewer behavioral problems, suggesting that hope may act as a protective factor against various challenges. In this context, the integration of hope into oral healthcare becomes particularly relevant. Feeling hope and being hopeful can offer a positive outlook and a sense of empowerment, especially in challenging situations. For example, hope has become recognized as a vital aspect of nursing care for individuals facing chronic or complex illnesses (20, 21). The study by Paramos et al. (20) provides a comprehensive list of interventions to foster hope and their corresponding evidence-based outcomes. One example highlighted in this study involves using honest and trustworthy explanations when working with adolescents with oncologic illnesses. The result of this intervention was a reduction in the levels of depression (22), showcasing the potential positive impact of hope-facilitating strategies. Drawing from Olsman's study (17), it becomes evident that cultivating strong relationship between healthcare providers and patient, characterized by compassion and empowerment (23, 24), not only enhances patient well-being but also significantly holds the potential to facilitate hope. This emphasizes the critical role of trust and positive relationships in fostering hope.

While there are many interventions to promote oral health in adolescents worldwide (25, 26) and in Norway (27–30), there has not been any prior research focusing on hope in this context. Hope has the potential to overcome the negativity surrounding dentistry and links well with the need to develop and deliver oral health interventions co-designed to ensure a patient-centered approach is employed. It is particularly relevant given the adoption of broad definitions of oral health and hope's beneficial effects on well-being demonstrated within the expanding field of positive psychology (31).

The World Dental Federation's (FDI) comprehensive definition of oral health emphasizes the profound interconnectedness of oral well-being with general health and overall quality of life (32). This holistic view underscores the importance of nurturing not only physical health but also emotional and psychological facets. Facilitating hope in the dental setting may give patients a sense of responsibility and empowerment to improve their oral health, which is currently lacking.

This paper presents findings from an ongoing project, #Care4YoungTeeth<3. Within the framework of this project, this study aimed to use observation to explore the approaches or techniques dental professionals use to communicate with adolescents aged 12–18 years, and then specifically identify examples that facilitate hope. This work was conducted to explore whether, in the future, an intervention to include hope-fostering techniques could be co-designed for use with adolescents by dental professionals.

## 2 Materials and methods

### 2.1 Context

In the realm of health and well-being, the salutogenesis theory, developed by Aaron Antonovsky in 1979, represents a profound shift from a disease-centric perspective towards a holistic focus on the health (33). This theory centers on the concept of “sense of coherence,” shaped by an individual's life experiences, which facilitates effective coping with stressors and determines their position on the health Ease/Dis-ease continuum. Salutogenesis delves into the positive aspects of human experience to comprehend how people maintain well-being, even in challenging conditions, akin to Antonovsky's river metaphor. He emphasizes that merely preventing stress is insufficient; individuals must also learn to swim for health promotion (34).

Hope, a positive aspect of human experience, actively involves individuals in maintaining well-being. Daily communications can contain many hopeful interactions and exchanges, contributing to a positive outlook and well-being (35). However, hope entails more than passive optimism; it involves a sense of responsibility and the willingness to put in effort (36). The importance of promoting hope becomes most apparent when considering the consequences of despair, particularly in the vulnerable age group of children and youth (36, 37). Adolescence, characterized by physical, psychological, social, and neurobiological shifts, signifies the vulnerable transition from childhood to adulthood in the second decade of life (38). Meanwhile, lower levels of hope have been observed in adolescents, with only older people reporting lower levels than this group (39). This suggests that there is a need to cultivate hope among adolescents and children for the purpose of health promotion.

Connecting the notion of health promotion to the theories of hope, particularly those of Snyder (40) and Plutchik (41), adds a multidimensional perspective to our understanding.

As defined by Snyder, hope has cognitive elements that include abilities to identify pathways to desired goals and the agency to utilize those pathways (40). A goal is defined as an envisioned outcome that individuals strive to achieve in the future. In the context of oral health, this means setting and working towards specific goals, such as maintaining clean teeth through regular brushing, attending dental check-ups regularly, and reducing sugar consumption. Pathways to achieving these goals require individuals to seek guidance, acquire new skills, and sustain motivation (42). Furthermore, incorporating agency into the narrative accentuates the importance of believing in one's capacity to act and achieve, which requires self-confidence, boundary establishment, and assumption of responsibility. For instance, in oral health, an agency might manifest as initiating a consistent oral care routine, with an example of a pathway being engaging with a dental professional.

Similarly, Plutchik stresses the emotional component of hope as a combination of anticipation and trust (43, 44). Again, these ideas can be readily applied to oral health and patient-dentist interactions.

The synergy of these theories signifies that hope is a cognitive-emotional construct intricately linked to one's goals, pathways, agency, trust, and anticipation.

The #Care4YoungTeeth<3 project is dedicated to enhancing adolescent oral health by co-designing interventions specifically developed by and for adolescents. By affording equal opportunities, adolescents can actively contribute as users, evaluators, informants, and co-designers, thereby considering their unique needs, curiosities, and social norms (45, 46). Embracing participatory research and design principles, the project adopts practice-based approaches and fosters multidisciplinary collaborations as its core strategies. These principles actively engage key stakeholders, including adolescents, dental practitioners, and caregivers, throughout all stages of the project. By incorporating their input, the aim is to ensure that the interventions are tailored to the needs and preferences of the target group, promoting their utilization and value among the adolescents they are designed to assist.

### 2.2 Research ethics

Before commencing the study, the project's research protocol received approval from the Norwegian Agency for Shared Services in Education and Research (Sikt). The study required written consent because personal data were being collected. Parents or caregivers provided consent for adolescents younger than 16 years, while those aged 16 years and older gave their own written consent. Three versions of information letters were created: one for adolescents above 16 years, one below 16 years, and one for parents and caregivers.

### 2.3 Research approach

Given the scarcity of research focused on oral health promotion aimed at this specific age group (47) and the need for a thorough understanding of communication and interaction (verbal and non-verbal) between adolescents and dental professionals, a qualitative research approach was chosen. Our set-up included observations of a dental consultation with subsequent interviews of the observed participants. This approach was employed to explore individuals' behaviors and personal perspectives, allowing for a comprehensive understanding of their practices and experiences. A variety of data collection techniques were utilized, encompassing video and audio recordings, photography, note-taking, and sketching, providing a novel method in dental research (9, 48).

The article is based on the data from four video observations, along with audio, pictures, notes, and sketches documenting consultations at two dental clinics. Video recording was chosen as an appropriate approach to record the real-time interaction between health professionals and patients (49). Additional materials like pictures, notes, and sketches were used alongside videos to help recall and reflect on observations. Documenting these condensed notes on the spot is considered highly valuable (50). Authors (KS, AJ) observed the consultations.

### 2.4 Participants

The first phase of the recruitment process in this study was inviting dental clinics in the region of Central Norway to take part by sending invitation letters by email to the heads of clinics. The participating dental professionals were thoroughly informed about the study. They received a separate written information and consent form, and written consent was obtained before their participation in the observation session and interview. They could ask questions about the study before signing consent and were thoroughly informed about the voluntariness of participating in the study. They performed the consultation with the adolescent as they usually do since we were interested in observing the usual interaction between dental personnel and adolescent (we are aware of the possibility of altered behavior when being observed); this means that the dental personnel did not receive any training or guidance on how and what to ask in this specific visit. However, most dental personnel in Norway received training in communication with adolescents and other patients [for instance, motivational interviewing (MI)] as part of their education or post-education training (51).

Thirteen clinics were invited in December 2021–September 2022, of which two consented to participate. Dental professionals at these two clinics sent information about the study as part of the standard invitation to the adolescents' regular check-ups. If the adolescent was under the age of 16, the letter was also sent to parents/caregivers. Adolescents and/or parents/caregivers were asked to call the clinic if they wanted more information about the study or if they were interested in participating. They were also informed that the clinic could call to ask for interest in taking part in the study. Dental professionals at the two clinics each identified two participants and communicated their contact information to the researchers, who provided further information if needed and obtained written consent.

The participants were chosen based on certain criteria. Participants for this study were adolescents aged 12–18 years old and had a planned regular visit in the period scheduled for data collection. Exclusion criteria included a history of no-show behavior, documented dental fear or anxiety, extensive dental treatment needs, limited communication skills, or personal familiarity with the researchers outside the dental clinic setting.

The two dental clinics participating in the study are in small towns in Central Norway. From each clinic, one girl and one boy (*n* = 4) participated, 12, 13, and 15 years old, while the participating dental professionals were one dentist and two dental hygienists (*n* = 3). All participants have been given pseudonyms (Appendix 1). No demographic information or medical history was collected by the authors during the observations.

For some adolescents, it was the first time they met the actual dental professionals at the visit we observed. In contrast, others had previously met the specific dentist/hygienist in previous visits. We know from interviews with the adolescents (results not shown in the paper) that they felt familiar with the dental professionals, even if this was the first time they met because they were familiar with the clinic.

The consultations observed were regular visits, usually lasting 20–30 min. Intervals between regular dental check-ups in Norway are between one and two years for children. However, if dental professionals suspect dental caries, gingival diseases, or other dental problems, the recall interval is shorter, and a new appointment for the patient is booked in the near future.

### 2.5 Analysis

After the observations, preliminary, substantive, and analytic reviews of videos were employed (49). The preliminary reviews were conducted shortly after the consultations (Appendix 1). Substantive reviews were initiated after watching the video recordings and familiarization with the key events and activities throughout the consultations. The recorded consultations were transcribed verbatim by one of the authors (KS), capturing verbal and non-verbal elements of dental consultations for subsequent analysis. A thematic analysis introduced by Braun and Clarke (52) was used. Authors (KS, AJ) initially watched the videos and read and reviewed the transcripts to identify thematic patterns illustrating the interactional facilitation of hope in dental consultations. A second round of collaborative, detailed watching and discussion of the videos was conducted by three authors (AJ, KS, MH). A deductive approach was then instructed to classify the data into themes in a shared document. Themes were clustered according to the characteristics of techniques employed. This was done as a collaborative and reflexive process (53) between authors (AJ). Eventually, determining the significance of the themes involved all authors.

## 3 Results

Our examination of the data identified three primary themes with associated sub-themes, shedding light on dental professionals' existing strategies and techniques, which inherently encompass crucial components of hope (Table 1). These techniques have the potential to instill a sense of hope among adolescents.

**Table 1:** Themes and sub-themes.

Theme	Sub-theme
Bonding strategies in dental consultations	1.1 Referring Back to Previous Visits
	1.2 Non-Judgmental and Empathetic Communication
	1.3 Empathetic Practices
Verbal and non-verbal strategies for creating positive relationships in dental consultations	2.1 Utilization of Positive Language
	2.2 Mitigating Negative Information or Bad News
	2.3 Non-verbal Strategies
Adolescents’ empowerment in dental consultations	3.1 Belief in Adolescents’ Capabilities
	3.2 Positive Reinforcement and Recognition
	3.3 Encouraging Patient Participation and Empowerment
	3.4 Indirect Advice and Shared Decision-Making

### 3.1 Bonding strategies in dental consultations

A central theme that emerged throughout the dental consultations was the dental professionals' seemingly intentional effort to build relationships with the adolescents. Dental professionals seemed to recognize the significance of the brief moments, starting from the waiting room and extending to the treatment room, to establish a meaningful relationship and foster trust with their young patients. Through a sense of continuity, non-judgmental and empathetic communication, dental professionals worked to create a supportive environment where adolescents could receive pathways and feel valued, understood, and hopeful about their dental care. The outcome of this connection-building effort could be a heightened sense of trust, leading to stronger patient-dentist relationships and enhanced hope for positive dental experiences in the future.

#### 3.1.1 Referring back to previous visits

Dental professionals referred back to the last time the adolescent had visited the clinic. This reference to previous encounters could contribute to a sense of coherence and familiarity, reinforcing the connection between the patient and the dental professionals.

The first thing Katie, the dentist, said to Shone (15-year-old boy): “*Has everything been going well since you were last here?*”

In another consultation, the hygienist Isabel referred back by saying: “*As we talked about last time, right, what we talked about last time, was that your tooth position is a bit- …*”

This approach of reconnections usually happened at the beginning of the consultations, building a bridge between the present moment and the previous time. This method serves as a transition to start the conversation and bond with adolescents.

#### 3.1.2 Non-judgmental and empathetic communication

Dental professionals maintained a non-judgmental stance, displaying empathy and understanding towards their adolescent patients. If a patient disclosed brushing habits that did not align with recommendations, dental professionals refrained from blaming or judging them: “*No, well, it’s perfectly fine if you forget to brush once in a while*” (Emily). In a consultation, hygienist Emily tactfully acknowledges 13-year-old Nathalie's choice to brush only in the evening. Rather than discrediting her, Emily praises this behavior, emphasizing its importance. She then suggested that if Nathalie could manage it, brushing in the morning would further strengthen her teeth.

Emily: “*mm, it is, as I said, brushing in the evening is in a way the most important, because then you kind of brush away [i.e., remove] everything that has come [i.e., bacteria] during the day, and then, if you leave the toothpaste [in the mouth] and let it work a little after brushing, that you kind of just brush and spit and go to bed, then the fluoride in the toothpaste strengthens your teeth a bit during the night. So, that [i.e., brushing in the evening] is the most important*”.

Nathalie: “Yeah”

Emily: “*And then, of course, if you manage to do it in the morning as well because it turns out that those who manage to brush and get fluoride on their teeth twice in a day, …*”

Nathalie: “*Yes*”

Emily: “*They get slightly stronger teeth than those who have once a day.*”

They empathized with the patients, acknowledging that occasional lapses were normal and offering pathways to improve oral care practices.

#### 3.1.3 Empathetic practices

Dental professionals demonstrated empathy in various ways. For example, they empathized with the discomfort associated with dental procedures like taking x-rays. They ensured that these processes were completed quickly to minimize any inconvenience, irrespective of whether the adolescent had explicitly stated discomfort/dread or not.

“*I know these are uncomfortable, so I tend to be quite quick.*” (Emily)

By recognizing the patients' apprehensions and reassuring them that their discomfort was understood, dental professionals could foster a trusting and compassionate environment.

### 3.2 Verbal and non-verbal strategies for creating positive relationships in dental consultations

A central theme that emerged throughout the dental consultations was verbal and non-verbal communication strategies for establishing a positive relationship. Through positive language, mitigating negative information, employing neutral language for describing challenges, and using welcoming non-verbal communication, dental professionals seemed to nurture an environment where adolescents could feel informed, supported, and confident in actively participating in their dental care decisions and taking charge of their oral health journey. These strategies could emphasize how they contribute to fostering adolescents' agency and gaining their trust during dental consultations.

#### 3.2.1 Utilization of positive language

During the consultations, dental professionals frequently employed positive language, using phrases and words like “*good*,” “*great*,” and “*perfect*.” This positive language was evident in various aspects of the interaction, such as praising apparently insignificant behavior during the consultation, like the adolescent's positioning of the head or issues beyond the adolescents' control, e.g., their occlusion or the condition of their oral mucosa, and assessing adolescents' accounts of their brushing habits.

“*I can see that you have brushed your teeth very well before you came*” (Isabel)

Specific positive assessments were given when acknowledging the adolescents' efforts, for instance, expressing appreciation for their diligent tooth brushing before the visit. This approach aimed to make adolescents feel supported and confident, ultimately fostering a sense of agency.

#### 3.2.2 Mitigating negative information or bad news

Dental professionals employed different approaches to deliver negative information. Euphemistic Language: (a) Dental problems such as calculus or erosion were referred to as, for instance, “*teeny tiny*”, i.e., employing euphemisms to soften the impact of negative information. (b) Positive Feedback before Bad News: The dental professionals seemed to strategically provide positive feedback or praise immediately before delivering potentially concerning news to help buffer the impact of negative information, for instance, when Isabel tells 15-year-old Clara that she has brushed her teeth really well today right before telling her she has gingivitis. (c) Neutral Language and Avoidance of Personal Pronouns: In contrast to the use of personal pronouns (“*you*”) for positive feedback [“*I could tell that you know a lot about acid erosion*” (Katie)], neutral pronouns or general phrases were used when discussing challenges or suggestions for improvements, such as “*most people*,” “*some*” *or* “*one*”, for instance “*we have to practice to be good at something*” (Emily) or “*it is an advantage to brush the teeth last thing at the night*” *(i.e., not* “*your teeth*”*, but* “*the teeth*”*)* (Katie). This approach conveyed that it is normal for adolescents to encounter learning curves and that practice is essential for improvement.

#### 3.2.3 Non-verbal strategies

Employing a holistic approach to non-verbal communication, dental professionals skillfully integrated various techniques to create a welcoming and engaging environment during consultations. Consistent eye contact conveyed attentiveness and connection, while enthusiastic smiles and varied tones of voice fostered positivity and rapport. Complementing these cues, open postures, whether seated or standing, could further establish a sense of trust and comfort. These non-verbal strategies seem key in encouraging adolescents to openly discuss their oral health experiences and concerns, promoting collaborative and effective healthcare interaction.

Common for the dental professionals in the observed consultations is that they complete certain tasks such as examination of the adolescents' mouth before they inform the patient or encourage them to talk. For instance, Isabel walks around in the consultancy room to put some equipment back, get a cup for Clara, and examine the x-ray images on the computer. She then moves back and sits down on the stool next to Clara and makes eye contact with her before initiating dialogue about diet, and Clara admits that she drinks a lot of energy drinks.

### 3.3 Adolescents' empowerment in dental consultations

During dental consultations, a prominent technique that emerged was the empowerment of adolescents. Through belief in adolescents' capabilities, positive reinforcement, participation, and shared decision-making, dental professionals not only could instill confidence but also encourage adolescents to approach dental care with a positive attitude. Empowering adolescents may bring about a shift in their perception of dental care, encouraging them to take an active role in maintaining their oral health. Within this approach, dental professionals could actively show a pathway and foster a sense of agency and confidence in their ability to take responsibility for their oral health journey.

#### 3.3.1 Belief in adolescents' capabilities

Dental professionals firmly believe in the adolescents' capacity to take care of their teeth effectively. They verbalized their confidence by indirectly stating, “*I believe in you*,” and offering adolescents the responsibility to make decisions about their oral health. By providing more than one suggestion and leaving it up to the adolescents to decide, dental professionals worked to empower them to play an active role in shaping their dental care routine and making informed choices about their oral health.

Pablo told the hygienist, Emily, that he rinses his mouth with water after brushing because he does not like the taste of toothpaste. Emily explains that if he rinses with water, his teeth may not get enough fluoride. She then suggests different options to ensure his teeth still get enough fluoride: testing different toothpaste to find one with an acceptable taste, taking fluoride tablets, or remembering to use fluoride mouth rinse daily. She leaves it up to him to decide.

#### 3.3.2 Positive reinforcement and recognition

Dental professionals expressed satisfaction and appreciation during consultations, applauding effective brushing and oral care practices. These actions might instill a sense of accomplishment and motivation in adolescents to continue their diligent oral care routines. Furthermore, dental professionals harnessed the power of encouragement and resilience-building. They reminded adolescents of past achievements, emphasizing the significance of these accomplishments rather than taking them for granted. By creating a supportive environment that celebrates their successes, adolescents might be encouraged to view oral care as a personal achievement worth valuing. Emily praised Pablo when he remembered the brushing technique she had presented to him earlier in the consultation (even if she had to give him a couple of hints to remember what she had said), and Katie became very enthusiastic when Shone told her that he had just started brushing his teeth in the morning and not just in the evening—“*that’s great!*”, she said, and added with an enthusiastic tone of voice that if he manages to brush in the morning every day for about three weeks, it will be established as a habit.

#### 3.3.3 Encouraging patient participation and empowerment

To foster a comfortable environment, dental professionals gave adolescents the opportunity to take breaks when needed, providing a “stop sign”, in this case, to raise one hand. The possibility to use a stop sign will also give the patient a possibility to contribute to the interaction even when not able to verbally articulate their needs and reduce the risk of feeling a loss of control. The stop sign could contribute to patient empowerment in a dental consultation.

#### 3.3.4 Indirect advice and shared decision-making

Instead of offering direct commands, the dental professionals used indirect approaches to give advice (pathway) and seek input from the adolescents: “*It is wise to have a system for brushing; do you have a system?*” (Emily). They gently asked questions about the patient's diet and habits, allowing them to self-reflect and arrive at their own conclusions.

Isabel: “*How about soft drinks and sweets and such?*”

Clara: “*I drink a lot of Red Bull then*”

Isabel: “*You drink Red Bull, yes. Aa. When you say a lot, what does that mean?*”

Clara: “*Like that.. a few times a week*”

Isabel: “*Yes, so it’s not just Saturday.*”

Clara: “*No*” (speaks very softly)

Isabel: “*No, it’s a bit more than that.*”

The patient nods.

This approach could help the patients feel empowered in making decisions about their dental health and encourage them to take ownership of their habits.

## 4 Discussion

The analysis of observations involving four adolescents and three dental professionals revealed that dental practitioners currently employ techniques conducive to fostering hope. This presents an opportunity for future interventions designed to explicitly nurture hope. This study explored the techniques, strategies, and methods three dental professionals used to communicate with four adolescents, then identified the elements related to the phenomenon of hope and how these elements seemed to be part of their current techniques. Three core themes and several sub-themes were identified from the observations: (1) Bonding strategies in Dental Consultations; (2) Verbal and Non-verbal Strategies for Creating Positive Relationships in Dental Consultations; and (3) Adolescents' Empowerment in Dental Consultations. These themes explain how dental professionals' endeavors to establish relationships create an environment where adolescents feel informed, supported, and confident in participating actively in their dental care decisions and managing their oral health journey. These techniques potentially deliver pathways, foster agency, cultivate trust, fortify patient-dentist relationships, and ultimately enhance hope for more positive future dental experiences.

Similar and other techniques were reported in behavior management and communication skills literature (54, 55). For example, Coolidge and Kotsanos (54) mentioned nonverbal communication like hand gestures and dental office atmosphere, providing written information before the visit, communication with parents, in addition to eyes, body posture, voice, verbal communication, and empathy. In the other study, Roberts et al. (55) introduced a wide variety of techniques that are universally accepted by pediatric and general dentists, like “desensitization,” “tell-show-do,” “modeling,” and “reinforcement.”

The adolescent phase represents a critical period for developing behaviors and habits that can significantly impact oral health outcomes (56). Establishing a strong foundation during adolescence is vital for preventing dental disease, addressing dental anxiety, and creating a positive dental experience. This foundation can serve as a cornerstone for adopting lifelong oral health behaviors. Adolescents and young children are particularly receptive to information and guidance during this formative stage when habits and behaviors are being shaped (57, 58).

The phenomenon of hope fits well with the principles of the salutogenesis theory. This approach encourages us to address beyond immediate treatment needs and consider individuals' overall well-being (59). For example, a study investigating the concept of a sense of coherence in mothers has shown an impact on children's attitudes toward dental procedures (60). This highlights the significance of adopting such holistic approaches in dental care. Moreover, it demonstrates the potential for *intentionally* fostering hope as an intervention to promote health, as the promising outcome (35). For example, introducing an intervention that helps adolescents identify small achievements can facilitate hope (61) in their ability to improve their oral health.

Motivational interviewing and hope share similarities in expressing empathy, fostering agency, and promoting resilience. Considering that only about half of the dental professionals who received MI training are confident in using it (51), hope might provide a structured framework for dental professionals. Our results suggest that elements of hope are already present in dental professionals' approaches to managing adolescent care, potentially strengthening the idea that hope is a relevant construct. Through a more thoughtful integration of hope into a dental practice via, for example, a co-designed intervention, the beneficial effects on adolescents' well-being could be extended. For instance, actively referring to goals, pathways, agency, and trust and discussing how to address these elements of hope from Snyder's and Plutchik's theories could improve adolescents' oral health literacy and potentially lead to oral health improvement. By virtue of their profession, dental professionals are important collaborators in realizing hope and being hopeful toward patients' positive outlook and well-being. Considering that adolescence is a critical phase of transition between childhood and adulthood and, as such, represents a vulnerable period (37, 38), it is essential to acknowledge the importance of intervening on multiple levels, including caregivers, schools, medical professionals, and policies (62).

However, it should be acknowledged that maintaining high levels of positivity and hopefulness would be challenging for those experiencing dental anxiety and with a history of extensive treatment. For these vulnerable groups or people in vulnerable circumstances, it is crucial to recognize the fear and apprehension often associated with dental visits. By emphasizing the potential for co-designed interventions tailored to these vulnerable populations' specific needs and experiences, we can work towards transforming their anxieties into positive dental experiences.

The extent to which dental professionals effectively facilitated hope remains somewhat unclear from our observations. Considering the potential benefits of this approach, we can contemplate establishing criteria for fostering hope in adolescents, which could have wider benefits for all patients. To support dentists in implementing these practices, a multifaceted approach is required. One avenue for intervention could involve setting clear goals for and with adolescents. Indeed, goal setting is one of the most used techniques for health behavior change (63). While this aspect was not explicitly evident in our current results, it appears that dentists may be delegating the responsibility of goal-setting to the adolescents themselves. An alternative strategy might involve collaborative goal-setting between dentists and adolescents, offering them multiple pathways to success tailored to their individual needs. This approach has the potential to be transformative, fostering hope and positive oral care practices among adolescents and patients more broadly. From a design perspective, the idea of “evidencing” (64), i.e., using visual and tangible communication to help people communicate and remember, would be valuable in the context of setting goals together. The future directions section delves deeper into these possibilities and sets the stage for future directions in our exploration of hope in dental health.

## 5 Strengths and limitations

The strengths of this study lie in its use of the theoretical framework, providing a solid foundation for understanding the complex phenomenon of hope. Additionally, using a novel research method implemented by a diverse, multi-disciplinary research team enhances the depth of the study's insights.

This study has some limitations. Firstly, contacting individuals within the healthcare sector, proved challenging, potentially introducing selection bias due to the inability to reach or include all intended participants.

Furthermore, despite concerted efforts to engage dental professionals, only two of 13 dental clinics accepted the invitation to participate. This restricted the sample size and potentially had an impact on the generalizability of the findings. The relatively small number of dental professionals raises concerns about fully representing this group's diversity of perspectives and practices.

Additionally, the study's sample size is limited as it comprises participants exclusively from Norway. To enhance the robustness of the findings, future research should aim for replication with a larger, randomly selected sample of adolescents, encompassing both those with dental anxiety and those without.

The interactions were in Norwegian; the translation and how to interpret them will have influenced the analysis in ways that are difficult to know. Yet, for observing non-verbal language, it is probably a strength that one of the observers did not have full proficiency in the Norwegian language.

The study primarily involved female dental professionals and did not include adolescents with dental anxiety since they were not recruited. Future studies could consider including a more diverse group of dental professionals and adolescents with anxiety for a broader perspective.

Dental professionals and adolescents knew that they were being observed during the sessions. They may have been prone to the Hawthorne effect.

## 6 Future directions

In the future, this work will be extended with incorporating interviews to gather insights into the experiences of adolescents in dental clinics and examining how the identified hope elements impacted these adolescents. Furthermore, an expansion of this work may involve the inclusion of perspectives from parents through interviews. Through integrating perspectives from both adolescents and parents/caregivers, the intention is to develop interventions to facilitate hope, thereby enhancing their pertinence and efficacy. Additionally, hope-facilitating interventions tailored to the distinct requirements of adolescents will be co-designed and tested.

## 7 Conclusion

In summary, this study investigated how dental professionals employ techniques during consultations with adolescents, uncovering elements associated with hope in their practices. Three key themes were identified. These themes illustrate how dental professionals' efforts to build relationships create an environment where adolescents can feel informed, supported, and confident in their dental care decisions, ultimately enhancing their sense of hope.

Adolescence is pivotal for shaping behaviors and habits significantly influencing oral health outcomes. Establishing a strong foundation during this period is critical for preventing dental diseases, addressing dental anxiety, and ensuring a positive dental experience, with implications for lifelong oral health behaviors.

As suggested by the results, fostering hope as an intervention can benefit adolescents' well-being. However, it is vital to recognize the challenges faced by individuals with dental anxiety and other circumstances. Tailored, co-designed interventions can transform anxieties into positive dental experiences.

While the extent of dental professionals’ effectiveness in facilitating hope remains somewhat uncertain, establishing criteria for fostering hope in adolescents and patients at large, along with collaborative goal-setting between dentists and adolescents, can offer avenues for future research. These strategies could impact hope and promote positive oral care practices among adolescents and patients, emphasizing the importance of multidisciplinary interventions and patient-centered care.

## Data Availability

Anonymized transcripts and notes that support the findings of this study are available from the corresponding author, upon reasonable request.
